# Recombinant protein production facility for fungal biomass-degrading enzymes using the yeast *Pichia pastoris*

**DOI:** 10.3389/fmicb.2015.01002

**Published:** 2015-09-23

**Authors:** Mireille Haon, Sacha Grisel, David Navarro, Antoine Gruet, Jean-Guy Berrin, Christophe Bignon

**Affiliations:** ^1^INRA, UMR 1163 Biodiversité et Biotechnologie FongiquesMarseille, France; ^2^Aix-Marseille Université, Polytech Marseille, UMR1163 Biodiversité et Biotechnologie FongiquesMarseille, France; ^3^Laboratory of Protein and Nucleic Acid Chemistry, The Rockefeller University, New YorkNY, USA; ^4^Architecture et Fonction des Macromolècules Biologiques, CNRS-Aix-Marseille University UMR 7257Marseille, France; ^5^INRA, USC 1408 AFMBMarseille, France

**Keywords:** recombinant proteins, expression platform, CAZymes, *Pichia pastoris*, factorial approach, automation, glycoside hydrolase

## Abstract

Filamentous fungi are the predominant source of lignocellulolytic enzymes used in industry for the transformation of plant biomass into high-value molecules and biofuels. The rapidity with which new fungal genomic and post-genomic data are being produced is vastly outpacing functional studies. This underscores the critical need for developing platforms dedicated to the recombinant expression of enzymes lacking confident functional annotation, a prerequisite to their functional and structural study. In the last decade, the yeast *Pichia pastoris* has become increasingly popular as a host for the production of fungal biomass-degrading enzymes, and particularly carbohydrate-active enzymes (CAZymes). This study aimed at setting-up a platform to easily and quickly screen the extracellular expression of biomass-degrading enzymes in *P. pastoris*. We first used three fungal glycoside hydrolases (GHs) that we previously expressed using the protocol devised by Invitrogen to try different modifications of the original protocol. Considering the gain in time and convenience provided by the new protocol, we used it as basis to set-up the facility and produce a suite of fungal CAZymes (GHs, carbohydrate esterases and auxiliary activity enzyme families) out of which more than 70% were successfully expressed. The platform tasks range from gene cloning to automated protein purifications and activity tests, and is open to the CAZyme users’ community.

## Introduction

Lignocellulosic biomass is recognized as a sustainable source of mixed sugars for producing second generation biofuels by fermentation, and for the synthesis of biomaterials (Lynd et al., 2008). In industry, saccharification of lignocellulose is performed enzymatically using fungal enzyme cocktails (Himmel et al., 2007). Due to the recalcitrant nature of lignocellulosic biomass, the development of more efficient fungal enzyme preparations is an active research field with the sequencing of hundreds of fungal genomes at the US Department of Energy Joint Genome Institute 1,000 fungal genome project, (Grigoriev et al., 2014). This wealth of genomic information opens new horizons and opportunities to improve the enzymatic conversion of biomass.

However, due to throughput differences the production of recombinant fungal proteins lags far behind that of data generated by –omic approaches, which creates a bottleneck in the process of unraveling the function of novel CAZymes. Although historically filamentous fungi (e.g., *Trichoderma reesei, Aspergillus niger*) and other yeast (e.g., *Saccharomyces cerevisiae, Yarrowia lipolytica*) have been used for expressing native or recombinant fungal enzymes (Dashtban et al., 2009), the yeast *Pichia pastoris* has become the premier example of yeast species used for the production of recombinant proteins (Cregg et al., 2000; Damasceno et al., 2012). Advantages of this yeast include the use of efficient and tightly regulated promoters, higher expression levels for lower cost than insect and mammalian cells. Compared to prokaryotic expression, it provides better protein folding, allows post-translational modifications (*N*- and *O*-glycosylation, methylation, and acylation), and is able to secrete high levels of recombinant proteins in the culture medium. In addition, it secretes very low levels of endogenous proteins none of which has been reported to be active on lignocellulosic biomass, which simplifies protein purification.

Finally, because filamentous fungi and yeast are phylogenetically close, *P. pastoris* is the organism of choice for expressing different classes of active fungal CAZymes, such as GHs (Bauer et al., 2006; Couturier et al., 2011a,b), CE (Topakas et al., 2010, 2012) and AA enzymes [i.e., lytic polysaccharide mono-oxygenases (Kittl et al., 2012; Bey et al., 2013)]. Having these many advantages over other expression systems makes *P. pastoris* an organism of choice for the production of industrial enzymes (Rabert et al., 2013).

Despite obvious advantages, we have found that heterologous expression of fungal enzymes in *P. pastoris* remained time-consuming, and reasoned that protocols for transformation, screening of transformants, cultures, purification of recombinant proteins could benefit from streamlining to try to increase protein production-throughput. In this study, we have used in-house fungal genes encoding CAZymes [GH5, GH11, GH45 (Couturier et al., 2011a,b)] that we previously successfully expressed in *P. pastoris* using pPICZα inducible vector, to establish a simpler standard protocol. In this paper, we have analyzed, and modified whenever possible, different steps of the protocol set-up by Invitrogen for expressing proteins in *P. pastoris* from the design of expression constructs to the assay of purified proteins, with the ultimate goal of setting-up a lab-scale recombinant protein production facility. This platform now makes the link between upstream expert annotation of CAZymes (www.cazy.org) (Lombard et al., 2014) and downstream automated assay of enzymatic activities (Lafond et al., 2012) and crystallogenesis (Sulzenbacher et al., 2002).

## Materials and Methods

### Enzymes

Taq (screening purpose) and Platinum pfx (cloning purpose) DNA polymerases (Invitrogen) were used in PCR experiments.

### Expression Constructs

Coding sequences to express were PCR amplified, and then inserted into Invitrogen vectors pPICZαA (Couturier et al., 2011a,b), pGAPZαA and pBGP1 (Sasagawa et al., 2011) (this study) inframe with N-terminal yeast α-secretion factor and C-terminal His_6_ tag encoding sequences. Plasmid pBGP1 was obtained by removing the Gateway cassette from pBGP1-DEST (Sasagawa et al., 2011) by XhoI/XbaI restriction digestion. The primers used were: GGGGCTAAAGAACTCGAGAAAAGAGAGGCTGAAGCTCTCCCCCAAGCACAAGGTG and TTTTCTAGACCCGCCGGGAGAGCATTGATAG for *Podospora anserine* GH5 (GenBank: HM357135.1), GGGGCTAAAGAACTCGAGAAAAGAGAGGCTGAAGCTGCCCCCGGTGAGCTTCCT and TTTTCTAGACCGTGTGTCTGGACATAAATGTC for *P. anserine* GH11 (GenBank: HM357137.2), and GGGGCTAAAGAACTCGAGAAAAGAGAGGCTGAAGCTGATGTCCCACTTTGGGGCCAATG and TCTAGACCTTCGTCCGTACGAGCACA for *P. pastoris* GH45 (GenBank: CAY71902.1). Except pBGP1, resulting recombinant expression plasmids were linearized with PmeI (pPICZαA) or AvrII (pGAPZαA) restriction enzymes.

### Preparation of Frozen Electrocompetent Cells and Transformation

Cells were grown as described by Invitrogen (easyselect_man.pdf) with the following modification: when OD_600_ was about 1.4, cells recovered from 250 ml culture were resuspended in 100 ml YPD supplemented with 20 ml 1 M HEPES pH8 and 2.5 ml 1 M DTT, and stored at 30°C for 15’. After washing as described in easyselect_man.pdf, they were eventually resuspended in 0.5 ml of cold 1 M sorbitol and 60 μl aliquots were frozen at -80°C.

Electroporation was performed as described in easyselect_man.pdf using 60 μl of thawed electrocompetent cells.

### Liquid Phase Selection and Expression Screening

#### General Strategy

Both were performed in 24-wells deepwell plates. Indicated volumes are for one well. An aliquot (200 μl) of sorbitol cell suspension was used to inoculate 2 ml YPD supplemented with 100 μg/ml Zeocin, which was then incubated for 3 days at 30°C under 225 rpm rotary shaking. This step has two aims: it selects transformed cells as do Zeocin-containing agar plates, and serves as a pre-culture for the following recombinant protein expression screening. The latter was performed by inoculating 5 ml Zeocin-free growth medium with 50 μl pre-culture and then incubating at 30°C under shaking. At the end of the expression period, culture supernatants were recovered by centrifugation for 5’ at 4000 *g* and their protein content analyzed by SDS-PAGE and/or enzymatic activity assay.

#### Constitutive Expression (pGAPZαA)

Protein expression screening was performed by inoculating 5 ml YPD with 50 μl pre-culture and then incubating for 4 days at 30°C under shaking.

#### Inducible expression (pPICZαA)

Protein expression screening was performed by inoculating 5 ml BMGY (easyselect_man.pdf) with 50 μl pre-culture, and then incubating overnight at 30°C under shaking. The plate was then spun as above and the cell pellet was resuspended in 1 ml BMMY (easyselect_man.pdf). The culture medium was daily supplemented with 3% methanol for 4 days.

### Automated Purification of Recombinant Proteins

#### Non-Automated Pre-Purification Steps

(i) At the end of the expression period, 24-wells deepwell plates were spun for 5’ at 4000 g to pellet the cells. Supernatants were transferred well to well to another 24-wells deepwell plate, and each well was supplemented with 1/10 supernatant volume of 250 mM Tris pH8. (ii) Two hundred microliters of a 50% suspension of Ni Sepharose^TM^ High Performance (GE Healthcare 17-5268-02) were loaded into each well of a 96-wells deepwell filter plate (AcroPrep^TM^ Advance 1 ml, 1 μm Glass Fiber, PALL PN 8131). (iii) 24-wells deepwell plates containing the supernatants were put on a TECAN Freedom Evo robot, and the 96-wells deepwell filter plate on the first manifold of the same robot (Navarro et al., 2010).

#### Automated Steps

Indicated volumes are for one well. The automate removed ethanol from Sepharose beads by vacuum aspiration for 30″ at -50 millibars (mb), and then added 200 μl buffer A (10 mm Tris pH7.8, 150 mM NaCl and 10 mM imidazole). After 2′, buffer A was eliminated by vacuum aspiration for 30″ at -50 mb. This step was repeated once. The robot transferred 800 μl of each culture supernatant from the 24-wells deepwell plate to each well of the 96-wells deepwell filter plate. After 3′ incubation, the supernatant was eliminated by vacuum aspiration for 60″ at -50 mb. If more than 1 ml supernatant was to be processed, this step was repeated the required number of times. At the end of the binding step, the robot washed the beads twice with buffer A as above, and then dried them by an additional 5″ -700 mb vacuum aspiration. The robot transferred the filter plate on top of a 96-wells microplate that had been put on the second manifold of the robot beforehand, and added 200 μl buffer B (10 mm Tris pH7.8, 150 mM NaCl and 500 mM imidazole) to each well of the filter plate. After 5′ incubation, the robot transferred the elution volume from each well of the filter plate to the corresponding well of the 96-wells microplate underneath by two successive vacuum aspirations for 20″ at -50 mb. Complete recovery of elution volume was achieved by performing a second vacuum aspiration for 5″ at -700 mb. The filter plate was then transferred back to the first manifold where beads were regenerated by performing the following operations. (i) Buffer B (200 μl) was added and after 2′ incubation removed by vacuum aspiration for 30″ at -50 mb. This step was repeated once. (ii) Buffer A (200 μl) was added and after 2′ incubation removed by vacuum aspiration for 30″ at -50 mb. (iii) Finally, beads were supplemented in 400 μl of 20% ethanol. The filter plate was sandwiched between two adhesive aluminum sheets (Dutscher reference 106524) and stored at 4°C.

### Enzymatic Activity Assays

Indicated volumes are for one well. GH5, GH11, and GH45 enzymatic activities were assayed by measuring the hydrolysis of 1% azo-glucomannan, azo-xylan, and azo-CM-cellulose (Megazyme), respectively. The assay is based on GH-dependent hydrolysis of a dyed substrate that produces low-molecular weight dyed fragments which remain in solution on addition of a precipitant solution. High-molecular weight material is removed by centrifugation and the color of the supernatant is measured. In practice, culture supernatants or purified enzymes (10 μl) were added to 50 μl of azo-substrate solution in 50 mM sodium acetate pH4.8 in 96-well plates. The latter were heat-sealed with aluminum sheet using the robot PlateLoc sealer, and incubated at 40°C for 2 h under 800 rpm rotary shaking. Ethanol (150 μl) was added to precipitate non-hydrolysed substrate. After 10′ at room temperature, plates were centrifuged at 4000 *g* for 5′. Supernatants were collected and their optical density measured at 590 nm. Activities were expressed as percentage of azo-substrate used. The 100% value was the OD_590_ of a well to which no ethanol and no enzyme had been added.

## Results and Discussion

In a previous work, three GH (GH5, GH11, and GH45) were successfully expressed in *P. pastoris* by strictly following the protocol proposed by Invitrogen (easyselect_man.pdf). Analysis of the original protocol resulted in a list of different steps that could be subject of optimization in terms of time, cost, or convenience. The list was made of: (1) using frozen competent cells instead of extemporaneously preparing competent cells; (2) using less plasmid for electroporation; (3) skipping the selection of recombinant clones on plate; (4) replacing tubes and flasks with deep-well plates in the screening process. In addition to simplifying the original protocol, the purification of secreted His-tagged proteins was automated to increase throughput.

### Expression Constructs

**FIGURE 1:**
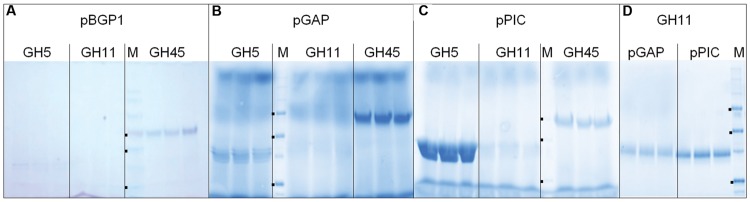
**Expression results of different DNA constructs. (A–C)**, 25 μl of culture supernatant using the indicated expression plasmid were directly analyzed by SDS-PAGE. **(D)** GH11 from **(B)** and **(C)** was purified by affinity chromatography on Ni-NTA, and 25 μl of elution fraction were analyzed by SDS-PAGE. M, molecular weight markers. **(A)** 180, 130, 100, 70 (■), 55 (■), 40, 35, 25 (■), 15, 10 kDa. **(B–D)** 250, 150, 100, 75 (■), 50 (■), 37, 25 (■), 20, 10 kDa.

In a first trial, GH5, GH11, and GH45 coding sequences were sub-cloned by restriction/ligation (R/L) into episomal pBGP1 (Sasagawa et al., 2011) and integrative pGAPZαA and pPICZαA expression vectors as described in Section “Materials and Methods,” and recombinant expression was evaluated by SDS-PAGE in the supernatant of culture media. Using integrative vectors was expected to result in genetically stable *P. pastoris* strains as they are integrated into the yeast chromosome at the cost of more laborious screening, whereas an episomal vector could be isolated by a simple plasmid preparation procedure. In addition, episomal vectors have higher transformation efficiencies and more than one copy per cell. It was therefore of interest to assess whether these features were correlated to recombinant protein expression level. Results are reported in **Figures 1A–C**. Expression of GH5 and GH45 was plasmid-dependant: GH5 was better expressed by pPICZαA than by pGAPZαA whereas it was the opposite for GH45. No GH11 was visible in the culture medium of any plasmid condition. To check whether GH11 was expressed, it was purified by affinity chromatography on Ni-NTA as described in Section “Materials and Methods” and analyzed by SDS-PAGE (**Figure 1D**). GH11 was slightly better expressed by pPICZαA than by pGAPZαA, although at levels definitely lower than those of GH5 and GH45. On the basis of above results, integrative vectors pGAPZαA and pPICZ were chosen for expressing fungal CAZymes. Two attempts to make DNA constructs easier resulted in negative expression results: the use of Gateway cloning instead of R/L (Additional File 1), and the use of PCR to both generate and linearize integrative expression constructs in a single experiment (Liu et al., 2008; Yu et al., 2012) (Additional File 2). In conclusion, the classical R/L technique followed by linearization by restriction of expression integrative constructs before transformation was used in the rest of this study, as proposed by Invitrogen (easyselect_man.pdf).

### Transformation

#### Frozen Electrocompetent Cells

Invitrogen advices not using frozen electrocompetent cells because freeze/thaw cycles reduce cell transformation efficiency. However, extemporaneously preparing electrocompetent cells is time consuming, which proportionally increases the cost price of recombinant proteins. The protocol used throughout this study and described in Section “Materials and Methods” was kindly provided by one of our collaborators (EIPL) and proved successful with all our expression constructs. No attempt was made to substitute electroporation with chemical transformation because the transformation efficiency was considered too low for the liquid phase transformation described below, although a recently launched chemical compound (BG2) seems promising in this respect (Filyak et al., 2013).

#### Amount of DNA for Transformation

As mentioned above, highest transformation efficiencies are not required in a recombinant protein expression facility because few clones are sufficient for expressing a recombinant protein. Since the number of individual colonies on agar plates is proportional to the amount of DNA used for transformation for a given transformation efficiency, we assessed the possibility to reduce the amount of DNA used for transformation. Invitrogen protocol suggests using 10 μg of linear expression construct per transformation. However, we found that as low as 0.5 μg of linear expression construct was enough for obtaining the necessary number of transformants for expressing recombinant proteins. Incidentally, using less DNA also allows using plasmid DNA mini-prep and not midi-prep kits, which is both faster and cheaper. An additional gain of time could be to skip the expression construct linearization by restriction, but we found that electroporating yeast cells with 0.5 μg of non-linearized integrative expression construct failed to give rise to colonies on agar plates (not illustrated). In conclusion, 0.5 μg of linear expression construct were decided to be, respectively, the default value and DNA state used on the expression platform.

#### Plating-Free Transformation

Invitrogen’s protocol implies plating cells on Zeocin agar plates after electroporation for selecting transformants. We reasoned that there were no reason why transformants could not be selected in liquid rather than in solid phase (i.e., on agar) provided the selection reagent Zeocin was included in the culture medium. Thereafter, we refer to selection of transformants by plating on Zeocin-containing agar plates as “solid phase” selection, and to selection of transformants by growing in YPD-Zeocin as “liquid phase” selection. The only weakness of the liquid phase option is that transformants are screened as a pool and not as individual clones. As a consequence, the expression screen result is the average of all clones that have been selected by the antibiotic, which can lead to suboptimal expression results.

**FIGURE 2:**
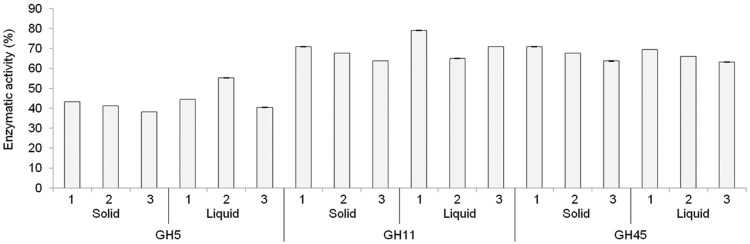
**Plating is dispensable for expressing recombinant GH in *Pichia pastoris*.** Liquid and solid phase selections were performed as described in the results section. For the solid phase selection, three individual clones (numbered 1, 2, 3 on the x axis) were grown each in triplicate in individual wells of a 24-wells deep-well plate. For the liquid phase selection, three individual wells (numbered 1, 2, 3 on the x axis) of a 24-wells deep-well were seeded each with 200 out of 1000 μl of the cell suspension in sorbitol solution after electroporation and assayed in triplicate. Results show the mean enzymatic activity assayed in culture supernatants, with standard deviations of the triplicate assay.

We compared both approaches to assess whether having individual clones was critical for our project. X33 cells were transformed with linearized pGAPZαA constructs bearing coding sequences for GH5, GH11, or GH45. After the sorbitol step, 200 μl of cell suspension in sorbitol were plated and then incubated at 30°C for 3 days as usual, and another 200 μl of the same cell suspension in sorbitol were used for liquid phase selection as described in Section “Materials and Methods”. When clones began to appear on agar, they were individually used to inoculate 5 ml YPD-Zeocin and then allowed to grow and express the recombinant protein for 4 days. Expression of recombinant proteins of all solid and liquid phase experimental points was assessed by assaying GH5, GH11, and GH45 enzymatic activities. Results (**Figure 2**) definitely show that, at least for our three reference proteins, there was no difference between either selection mean. In addition to using less Zeocin than solid phase selection [2 μl in 2 ml YPD instead of 25 μl in 25 ml YPD containing 1.2% agar (YPDA; i.e., the volume poured per plate)], liquid phase selection does not preclude individual clones to be selected on plate if necessary since it uses only 200 out of 1000 μl of the sorbitol suspension of transformed cells and so an additional 200 μl aliquot can be plated and grown in parallel with the liquid phase selection. In conclusion, the liquid phase selection was chosen as default option for our facility because it allows a yes/no answer to be obtained more quickly than solid state screening. In case of negative or poor response, plated clones can be used as a rescue.

### Culture of Transformants

#### Culture Dish

Based on our experience in recombinant expression screening in *Escherichia coli* (Noguere et al., 2012) and in filamentous fungi (Alberto et al., 2009), the ANSI/SLAS (American National Standards Institute/Society for Laboratory Automation and Screening) format was preferred to classical tubes and flasks for growing cells. This format has two advantages. First, dishes stand alone and do not require additional equipment such as tube or flask holders, which is convenient when many samples have to be processed in parallel as it is often the case on a dedicated platform. Second, it was devised for use with automates, which allows for a direct transfer from culture incubators to robot benchwork.

Although 96-wells deepwell plates have been successfully used for growing and co-expressing recombinant proteins in *P. pastoris* (Geier et al., 2013), 24-wells deepwell plates better fitted with the requirements of this type of platform. By accommodating larger culture volumes than 96-wells deepwell plates (5 instead of 1 ml per well), 24-wells deepwell plates act as a magnifying tool of recombinant protein expression for the following reason. Since recombinant proteins secreted by *P. pastoris* in the culture medium are C-terminally His6-tagged, they can be concentrated up to about 25 times by collecting them by affinity chromatography on Ni-NTA beads if elution from the affinity column is performed using 200 μl elution buffer (see further). Hence, even tiny levels of expression can be detected by SDS-PAGE analysis and Coomassie blue staining. By contrast, each well of a 96-wells deepwell plates provides five times less signal on gel for the same expression level because it can accommodate only 1 ml of culture medium. In addition, since oxygen transfer in 24-wells deepwell plates is higher than in 96-wells deepwell plates (Duetz, 2007), one may expect higher expression levels in the former than in the latter. Baﬄed 16-wells deepwell plates used for growing filamentous fungi (Alberto et al., 2009) were eventually not considered for use on the platform because the number of wells was too small for the intended screening throughput and their geometry unfitted for the automate six needle pipetting arm. In conclusion, 24-wells deepwell plates were chosen as default culture dish.

#### Managing Zeocin Consumption

Yeast expression plasmids of pGAPZ and pPICZ series confer Zeocin resistance to transformed cells, which allows for easy selection of the latter after plating on Zeocin-containing agar plates. However, Zeocin has two failings: it is expensive and degrades rapidly with time. In particular, it is light sensitive. Therefore, Zeocin-containing agar plates cannot be stored for long periods of time before use and must be protected from light throughout the process ranging from pouring to plating, which makes their handling inconvenient.

We addressed the issues of cost and degradability by testing the possibility of directly adding Zeocin to suspensions of electroporated cells in sorbitol solution rather than to agar plates.

**FIGURE 3:**
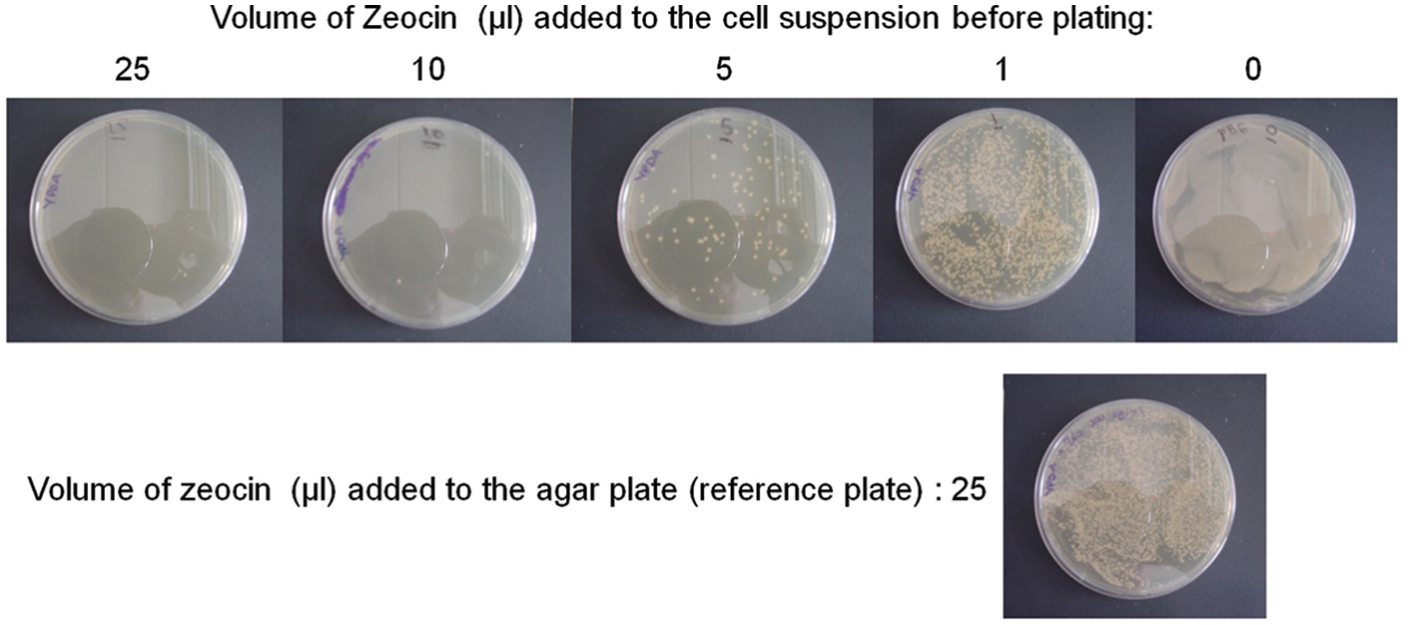
**Managing Zeocin consumption.** The experiment was performed as described in the results section. After 4 days at 30°C, plates were photographed. In the upper row, Zeocin was added directly to the cell suspension before plating on Zeocin deficient plates. Numbers on top indicated the volume of Zeocin mother solution added to 150 μl of cell suspension. The lonely plate below the upper row is the reference plate. In that case, the cell suspension was not supplemented with Zeocin, but was plated on Zeocin-containing plate. This reference plate was located between plates “0” and “1” of the upper row because it roughly contained an intermediate number of clones between these two plates.

In practice, X33 cells were electroporated with episomal Gateway plasmid pBGP1-DEST bearing GH5 coding sequence. After the sorbitol step, 100 out of 1000 μl cell suspension were spread on either a Zeocin plate made of 25 ml of YPDA supplemented with 25 μl of 100 mg/ml Zeocin solution, or on agar plates made of 25 ml of Zeocin-free YPDA. In the latter case, cell suspensions were supplemented with 0, 1, 5, 10, or 25 μl of 100 mg/ml Zeocin solution immediately before spreading on Zeocin-free plates. Plates were then incubated at 30°C for 4 days, and the number of colonies was compared. Results (**Figure 3**) indicate that adding 1 μl of Zeocin solution directly to the cell suspension just before plating provided roughly the same result as plating the same volume of the same cell suspension on an agar plate supplemented with 25 μl of Zeocin solution.

Adding Zeocin to the cell suspension immediately before plating rather than in the agar plate allows (i) dividing Zeocin consumption by about 50 for the same selection efficiency, (ii) preparing YPDA plates in advance of use and then storing them without particular caution except asepsis. In conclusion, if the liquid phase selection described in a previous paragraph cannot be performed because isolating clones is mandatory for some reason, this can be done at lower cost and more conveniently by adding Zeocin to the cell suspension just before plating rather than in the YPDA plate.

#### Prevention of Bacterial Contamination

Because integrative plasmids are generally used for transforming *P. pastoris* cells for which maintaining antibiotic selection pressure after transformants have been selected is unnecessary, and because the antibiotic used to select transformants (Zeocin) is expensive, no antibiotic is used for growing clones thereafter. Sometimes, however, the absence of antibiotic in rich culture media such as YPD allows bacterial contaminations to occur with the mandatory loss of the contaminated batch. Penicillin and Streptomycin are routinely used to prevent bacterial contaminations in eukaryotic cell line cultures. Being eukaryotes, yeasts should not be sensitive to antibiotics commonly used by microbiologists (ampicillin, chloramphenicol, kanamycin, tetracycline), which could thus be added to the culture medium without deleterious effect while preventing such contaminations.

**FIGURE 4:**
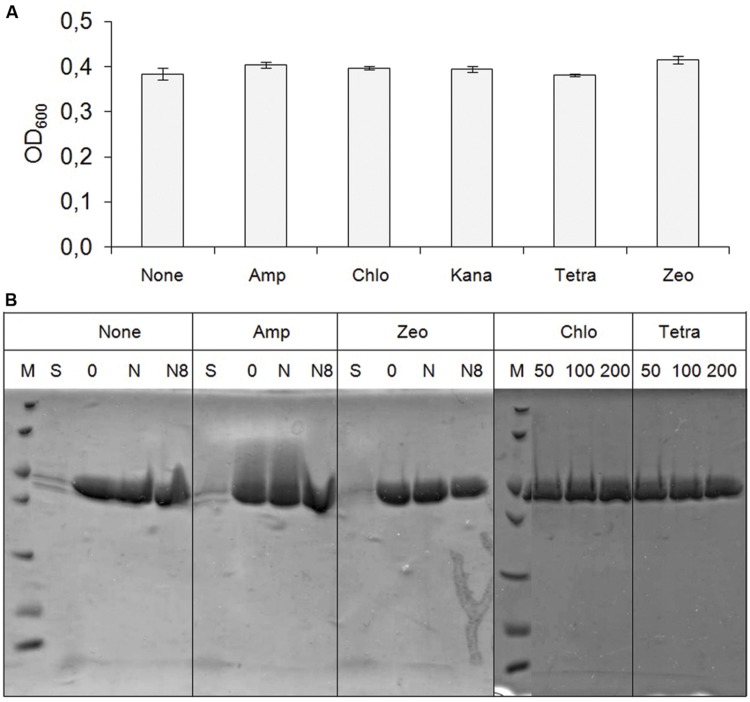
**Antibiotics have no effect on *P. pastoris* growth nor on protein expression. (A)** Effect of antibiotics on cell growth. The y axis is the optical density of a 1/300 dilution of the culture. None, no antibiotic; Amp, 100 μg/ml ampicillin; Chlo, 34 μg/ml chloramphenicol; Kana, 50 μg/ml kanamycin; Tetra, 15 μg/ml tetracycline; Zeo, 100 μg/ml Zeocin. **(B)** Recombinant proteins purified by affinity chromatography on Ni-NTA beads from 4 ml culture. From 100 μl of elution volume, 15 μl were analyzed by SDS-PAGE along with 15 μl of non-purified supernatant (lanes S). M, molecular weight markers; from top to bottom: 116, 66.2, 45, 35, 25, 18.4, 14.4 kDa. Numbers 50, 100, 200 in lanes Chlo and Tetra indicate the volume in μl of 50% Ni-NTA beads suspension used. See results for details.

To check that antibiotics had really no effect on yeast growth, a clone of *P. pastoris* cells expressing GH5 from pGAPZαA was grown for 4 days in YPD in the presence or absence of the antibiotics listed above at concentrations used in laboratories to select resistant *E. coli*. Biomass was assessed by measuring the optical density of the culture at 600 nm at the end of that period, which definitely demonstrated the total innocuousness of this series of antibiotics (**Figure 4A**). The amount of recombinant protein secreted in the culture medium was next compared. To that end, His6-tagged GH5 was concentrated by affinity chromatography on Ni-NTA beads under different conditions that are detailed in the next paragraph. Results (**Figure 4B**) showed no difference between protein expression in the presence or absence of antibiotic, suggesting that antibiotics have no negative effect on the over expression of a recombinant protein in *P. pastoris*. In conclusion, if bacterial contaminations are an issue commonly used antibiotics can provide good protection at much lower cost than Zeocin, and without effect on *P. pastoris* cell growth or protein expression.

### Protein Purification

Recombinant proteins were expressed as fusion proteins with an N-terminal signal peptide and a C-terminal His6-tag. This combination has two consequences. First, recombinant proteins are secreted in the culture medium as are biomass-degrading enzymes by filamentous fungi in their native environment. Second, this diluted protein solution can be concentrated up to about 25 times (compare, for example, lanes S to any other lane in **Figure 4B**) by collecting tagged proteins by affinity chromatography on Ni-NTA beads. The latter has two requirements: high specific binding is favored by slightly alkaline pH (about 8), and low non-specific binding requires high salt concentrations. Since YPD is devoid of any added salt and its pH ≈6, the addition (**Figure 4B**) of 0.3 M NaCl with (lanes N8) or without (lanes N) 100 mM Tris pH8 was compared to no addition (lanes 0). Results indicate that such additions were not required. However, when the culture medium pH is willingly maintained acidic (pH3) as in the fractional factorial approach described in the next paragraph, buffering using a final concentration of 100 mM Tris pH8 is mandatory before binding. The amount of Ni-NTA beads proved not limiting since increasing them from 50 to 200 μl of a 50% beads suspension did not increase the recovery yield (**Figure 4B**, lanes Chlo and Tetra).

**FIGURE 5:**
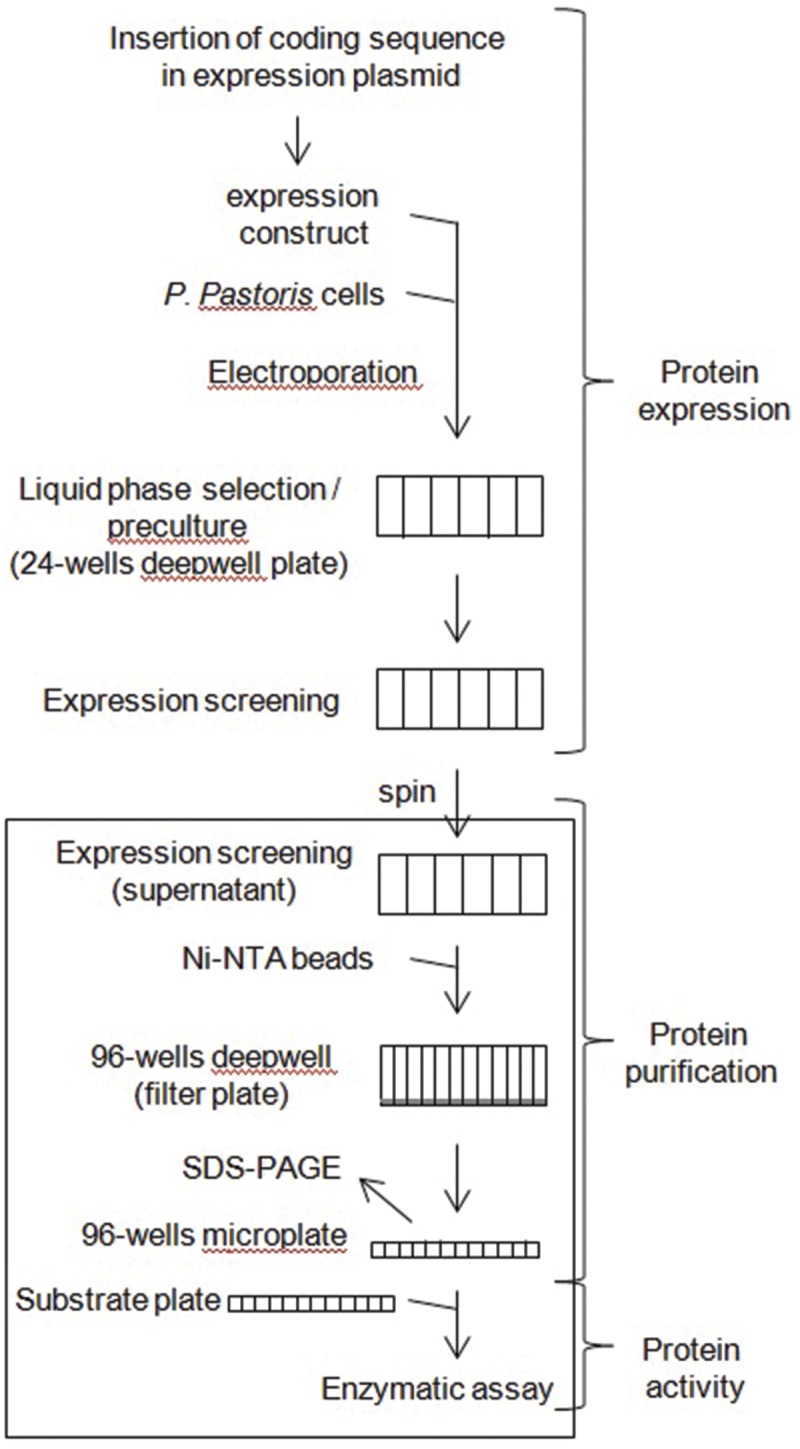
***Pichia pastoris* recombinant protein expression platform.** Automated purification by affinity chromatography on Ni-NTA and enzymatic activities assays of His6-tagged recombinant proteins are boxed. See Section “Materials and Methods” for details.

**FIGURE 6:**
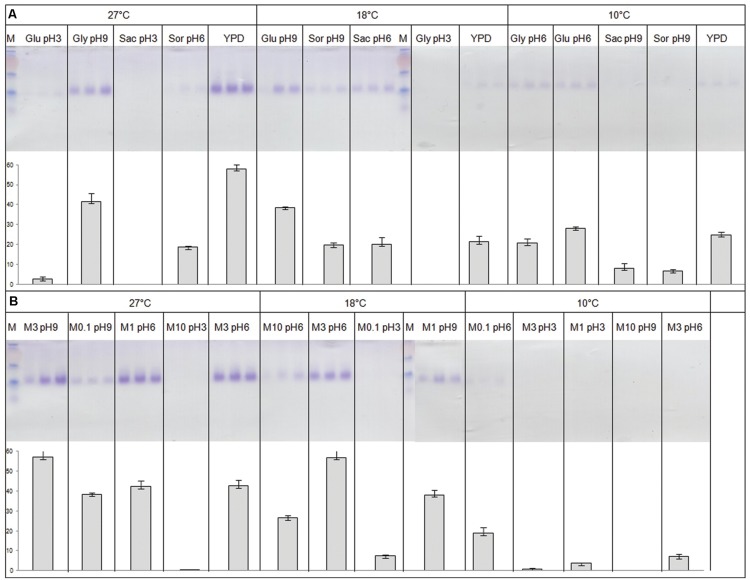
**Constitutive and inducible expression of a recombinant protein in *P. pastoris* using fractional factorial approaches.** Recombinant GH11 was purified by affinity chromatography on Ni-NTA as described in Section “Materials and Methods” and in **Figure 5**, and analyzed by SDS-PAGE and enzymatic assay. **(A)** Constitutive expression using pGAPZαA. **(B)** Inducible expression using pPICZαA. Parameter value combinations of the fractional factorial approach are indicated on top of each experimental point. Glu, Glucose; Gly, Glycerol; Sac, Saccharose; Sor, Sorbitol. M0.1, M1, M3, M10 stand for 0.1, 1, 3, 10% methanol concentration, respectively. M, molecular weight markers (same as **Figure 1A**; GH11 migrates as the 40 kDa band). See Additional File 3 for details.

Since deepwell plates can be directly transferred from a shaking incubator to a robot benchwork, the recovery of His6-tagged recombinant proteins by affinity chromatography on Ni-NTA was automated. To that end, Ni-NTA beads were used in deepwell filter plates as described in Section “Materials and Methods.” Note that 100 μl of beads per well were used because using 50 μl provided less reproducible results (not illustrated). The flowchart of the automated process is illustrated by **Figure 5**, and a thorough description of the robot can be found in (Navarro et al., 2010). After elution, recombinant His6-tagged proteins were analyzed by gel electrophoresis using pre-cast gels, and by assaying their enzymatic activity (**Figure 6**).

### Expression Conditions

It is our experience that modifying expression parameters in *E. coli* by trial and error is less effective than testing the same parameters in a factorial approach, mainly because parameter interaction is lost in the former case (Benoit et al., 2007; Noguere et al., 2012). Therefore, GH11 expression was tested in two different fractional factorial approaches using pGAPZαA and pPICZαA vectors, respectively. A detailed description of the method and of the results is in Additional File 3. In short, both approaches converged to the conclusion that expression was proportional to the temperature, and that an alkaline pH was more favorable than an acidic one (**Figure 6**).

## Conclusion

The recombinant protein production facility using *P. pastoris* should avoid using episomal and/or Gateway vectors because the former uselessly request maintaining expensive Zeocin selection throughout the expression process, and the latter failed at efficiently expressing three reference proteins. We have observed that expressing three reference CAZymes was plasmid-dependent. However, since testing both constitutive and inducible expression in parallel might prove impractical for large projects, a serial approach is more realistic. In case of negative result, swapping plasmids is made easy by the identity of cloning regions and reading frames of both vectors. Expression constructs should better be made using classical R/L followed by linearization by restriction. However, large projects may require using synthetic genes with yeast-optimized codons. After electroporation, transformants should be selected with Zeocin in liquid phase as a pool and not as individual colonies on agar plates. However, since electroporated cells can be stored for weeks at 4°C as sorbitol suspensions and only 200 μl out of 1000 are used for liquid phase selection, another 200 μl aliquot should be plated in parallel to later screen for higher producers (i.e., bearing gene multi-copies) if required. In addition, in case of bacterial contamination it is possible to start a new culture from liquid phase selection pre-culture (i.e., without the need for another electroporation) in the presence of one or more of the anti-microbial antibiotics tested in the present study. For large projects, basic expression conditions (standard culture media, 27°C) are preferred in first intention. By contrast, fractional factorial approaches may be performed in case of single protein project low expression yields and for high-added value proteins. In the ANSI/SLAS format, 24-wells deepwell plates are used for growing yeasts, liquid phase selection of transformants and expression screening. Recombinant proteins secreted in the culture medium are purified and concentrated by automated affinity chromatography on NiNTA beads.

**FIGURE 7:**
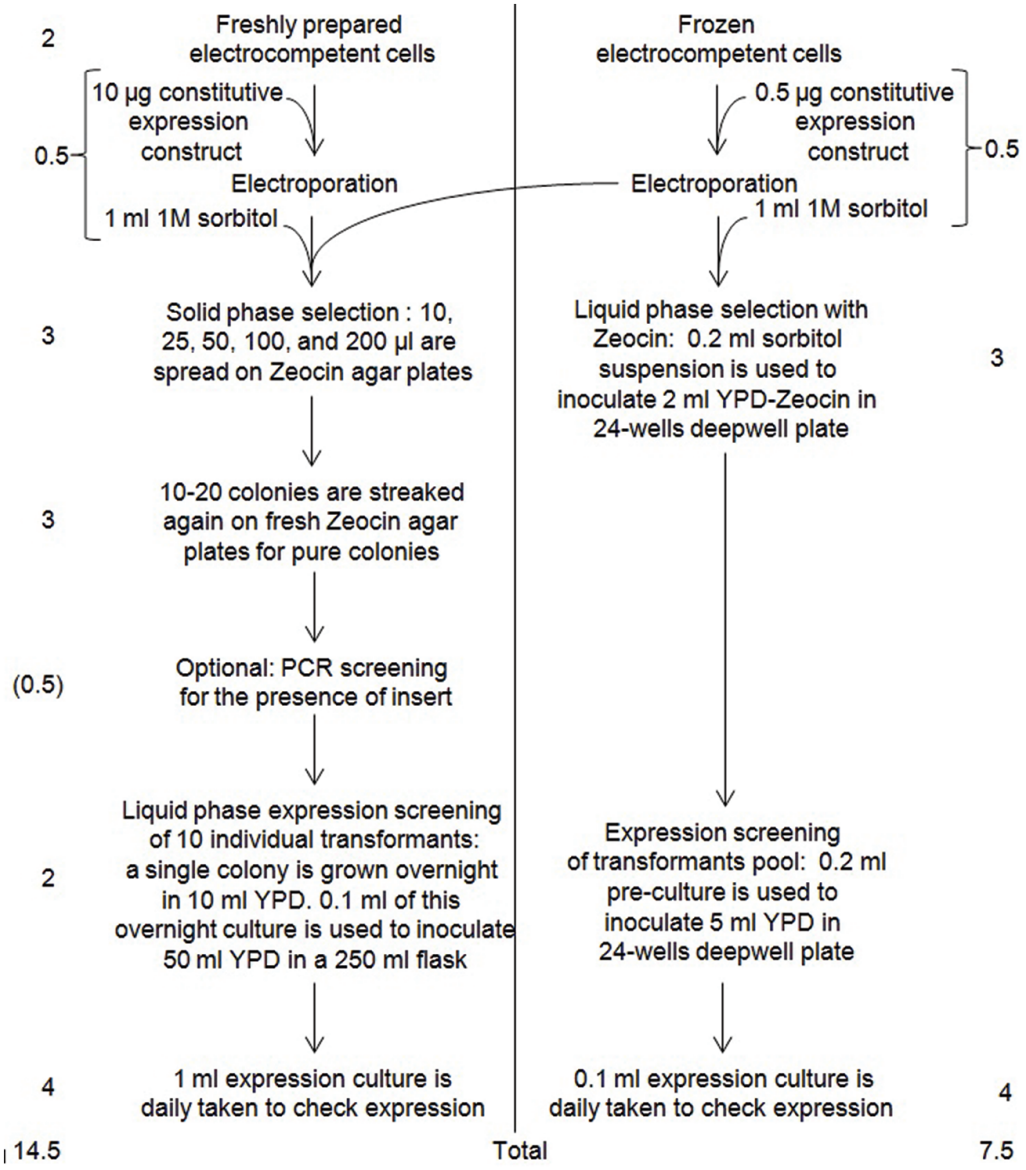
**Comparative flowchart of Invitrogen **(left)** and our **(right)** protocol for expressing recombinant proteins in *P. pastoris*.** In this example, constitutive expression was chosen. Numbers on the left and on the right are the estimated number of days required by the considered step, with their sum (total) at the bottom.

A comparative flowchart of the classical protocol set-up by Invitrogen (pgapz_man.pdf) and of the simplified protocol described here for expressing biomass-degrading fungal enzymes is provided in **Figure 7**. Overall, the simplified protocol allows dividing by two the time required to go from transformation to expression screening, and was used as a basis for setting-up a recombinant protein production facility for the heterologous expression of fungal CAZymes in *P. pastoris* (**Figure 5**). **Table 1** summarizes all the results obtained using *P. pastoris* as host for expressing fungal biomass-degrading enzymes in replacement of native or recombinant expression in other fungi such a *A. niger* and *T. reesei* (Levasseur et al., 2004; Poidevin et al., 2009). Switching from the original Invitrogen protocol to the simplified protocol described in this report and now routinely used on the platform has resulted in an increased use of recombinant expression in yeast from a few coding sequences in 2011 to a total of more than 40 today, out of which more than 50% were expressed at high level (>100 mg/L). The fungal CAZymes functionally produced originate from several filamentous fungi and belong to different CAZy families within the GH, CE, and AA classes. The platform is accessible to the community of CAZyme users upon request.

**Table 1:** The different enzymes expressed on the platform are reported.

Fungal strain	CAZy family	CBM	Predicted activity	Genbank ID	cDNA (bp)	Produced	Yield	Reference
*Ustilago maydis*	GH3	–	β-glucosidase	3634215	2619	No	–	–
*Podospora anserina*	GH5	–	β-1,4 mannanase	6194921	1236	Yes	>100	Couturier et al., 2011a
*P. anserina*	GH5	–	β-1,4 mannanase	6187561	1388	No	–	–
*P. anserina*	GH6	1	Cellobiohydrolase	6187194	1544	Yes	>500	Poidevin et al., 2013
*P. anserina*	GH6	–	Endoglucanase	6187311	1319	Yes	>500	Poidevin et al., 2013
*P. anserina*	GH6	1	Cellobiohydrolase	6188027	1258	Yes	>500	Poidevin et al., 2013
*P. anserina*	GH10	1	β-1,4 xylanase	6188644	1387	No	–	–
*P. anserina*	GH11	–	β-1,4 xylanase	6197075	742	No	–	–
*P. anserina*	GH11	1	β-1,4 xylanase	6187330	1060	Yes	<100	Couturier et al., 2011a
*P. anserina*	GH26	35	β-1,4 mannanase	6188179	1466	Yes	>100	Couturier et al., 2011a
*P. anserina*	GH27	–	α-galactosidase	6195125	1370	No	–	–
*U. maydis*	GH27	35	α-galactosidase	3632217	1719	No	–	–
*P. anserina*	GH43	–	β-xylosidase	6187343	1629	No	–	–
*Pichia pastoris*	GH45	1	β-1,4 glucanase	8201351	1845	Yes	>500	Couturier et al., 2011b
*P. anserina*	GH51	–	α-L-arabinofuranosidase	6188578	2251	Yes	>100	Couturier et al., 2011a
*U. maydis*	GH51	–	α-L-arabinofuranosidase	3628887	2016	Yes	>100	unpublished
*U. maydis*	GH62	–	α-L-arabinofuranosidase	3632410	996	Yes	>100	Siguier et al., 2014
*P. anserina*	GH62	–	α-L-arabinofuranosidase	6188704	1068	Yes	>100	Couturier et al., 2011a; Siguier et al., 2014
*P. anserina*	GH67	–	α-glucuronidase	6189855	2591	No	–	–
*P. anserina*	GH93	–	arabinanase	6188440	1274	Yes	>100	unpublished
*P. anserina*	GH131	1	β-1,4 glucanase	6187659	1093	Yes	>500	Lafond et al., 2012
*Talaromyces funiculosus*	CE1	1	Cinnamoyl esterase	AJ291496	1059	Yes	n.d.	unpublished
*Penicillium purpurogenum*	CE1	1	Acetyl xylan esterase	AF529173	1146	Yes	n.d.	unpublished
*Volvariella volvacea*	CE1	1	Acetyl xylan esterase	ABI63599	1047	Yes	n.d.	unpublished
*Pycnoporus cinnabarinus*	CE1	1	Carbohydrate esterase (CE)	CDO72503	1128	Yes	n.d.	unpublished
*P. anserina*	CE15	1	Methylglucuronic esterase	6187208	1446	Yes	<100	Katsimpouras et al., 2014
*P. anserina*	CE15	–	Methylglucuronic esterase	6189833	1188	Yes	<100	unpublished
*P. anserina*	CE16	1	Acetyl esterase	6189799	1329	Yes	<100	unpublished
*U. maydis*	AA3_2	–	GMC oxidoreductase	3632124	1911	Yes	>500	unpublished
*U. maydis*	AA3_2	–	Glucose oxidase	3631645	1905	Yes	>100	unpublished
*P. anserina*	AA3_2	–	Cellobiose dehydrogenase	6188032	2376	Yes	>500	Bennati-Granier et al., 2015
*Pycnoporus cinnabarinus*	AA3_2	–	Cellobiose dehydrogenase	AF081574	4500	Yes	>500	Bey et al., 2011
*U. maydis*	AA5_1	–	Glyoxal oxidase	3629211	1959	No	–	–
*U. maydis*	AA5_1	–	Glyoxal oxidase	3630482	2589	No	–	–
*P. anserina*	AA9	1	Lytic polysaccharide monooxygenase	6195934	1056	Yes	>100	Bey et al., 2013
*P. anserina*	AA9	1	Lytic polysaccharide monooxygenase	6192283	1043	Yes	>500	Bey et al., 2013
*P. anserina*	AA9	–	Lytic polysaccharide monooxygenase	6192256	783	No	–	–
*P. anserina*	AA9	–	lytic polysaccharide monooxygenase	6190208	1038	Yes	>100	Bennati-Granier et al., 2015
*P. anserina*	AA9	1	Lytic polysaccharide monooxygenase	6191260	934	Yes	<100	Bennati-Granier et al., 2015
*P. anserina*	AA9	–	Lytic polysaccharide monooxygenase	6195000	902	Yes	<100	Bennati-Granier et al., 2015
*P. anserina*	AA9	–	Lytic polysaccharide monooxygenase	6196037	778	Yes	<100	Bennati-Granier et al., 2015
*P. anserina*	AA9	1	Lytic polysaccharide monooxygenase	6187700	1113	Yes	>100	Bennati-Granier et al., 2015

*Yield, amount of secreted recombinant protein (mg/L).*

## Author Contributions

MH performed most of the experiments.

SG set up and optimized NiNTA protein purification protocols in collaboration with DN who was more involved in the automation thereof.

AG was in charge of factorial approaches.

J-GB had the idea of setting-up a recombinant protein expression facility using *P. pastoris* and proposed that CB be in charge of its set-up. He also provided several guidelines and participated in the writing of the manuscript.

CB preliminarily tested different experimental options developed in this study, coordinated the effort of above people and wrote the paper.

## Conflict of Interest Statement

The authors declare that the research was conducted in the absence of any commercial or financial relationships that could be construed as a potential conflict of interest.

## Abbreviations

AA: auxiliary activities
CAZy: carbohydrate-active enzymes
CE: carbohydrate esterase
GH: glycoside hydrolase
kDa: kiloDalton
SDS-PAGE: sodium dodecyl sulfate polyacrylamide gel electrophoresis
