# Supplementary material for: Recombinant protein production facility for fungal biomass-degrading enzymes using the yeast Pichia pastoris
Source: Front Microbiol. 2015 Sep 23;6:1002. doi: 10.3389/fmicb.2015.01002 (PMC4585289; doi:10.3389/fmicb.2015.01002)
Supplement: Supplementary file 1 [file Data_Sheet_1.DOCX]

**Additional file 1: Gateway cloning generates non expressing *P. pastoris* clones**

On the basis of published reports [1, 2], we tested Gateway technology for making expression constructs.

**Methods.**

For comparing Gateway and R/L cloning techniques, GH5 GH11, and GH45 coding sequences were PCR amplified using either primers containing Gateway recombination sites attb1 and 2 (ggggacaagtttgtacaaaaaagcaggct*CA*CTCCCCCAAGCACAAGGTG and

ggggaccactttgtacaagaaagctgggtAAGCCGGGAGAGCATTGATAG for GH5, ggggacaagtttgtacaaaaaagcaggct*CA*GCCCCCGGTGAGCTTCCT and

ggggaccactttgtacaagaaagctgggtAAAGGCACTGGGAATACCACTG

for GH11, ggggacaagtttgtacaaaaaagcaggct*CA*GATGTCCCACTTTGGGGCCAATG and

ggggaccactttgtacaagaaagctgggtATTCGTCCGTACGAGCACAGC for GH45) or primers containing XhoI and XbaI restriction sites (see Methods) at their 5’ end. The former PCR products were cloned into the episomal Gateway plasmid pBGP1-DEST [2] by BP and LR reactions. The latter PCR products were cloned into the episomal non-Gateway plasmid pBGP1 [3] by restriction / ligation. Plasmid pBGP1 was obtained by removing the Gateway cassette from pBGP1-DEST by XhoI / XbaI restriction digestion.

**Results and discussion.**

The coding sequences of GH5, GH11, GH45 [4, 5] were individually inserted into episomal yeast expression vectors as described in Methods. *P. pastoris* X33 cells were electroporated with the same amount of construct, and transformants were selected on Zeocin agar plates. All constructs provided comparable numbers of clones (more than 5,000 per µg of DNA; data not shown), suggesting that *P. pastoris* transformation efficiency is not affected by the cloning method neither by the size of the insert (respectively 1020, 770, and 960 bp).

Expression of recombinant proteins secreted in the culture medium was then assessed by gel electrophoresis. Results are illustrated in Figure 1.


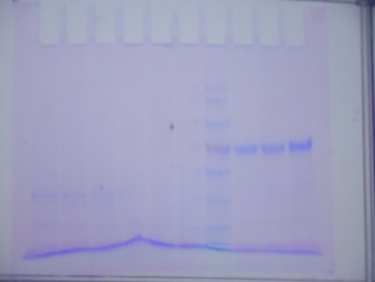


GH5

GH11

GH45


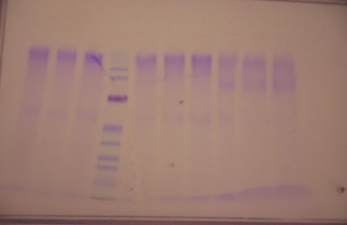


GH5

GH11

GH45

pBGP1-DEST

M

M

pBGP1

*

*

**Figure 1. Expression results of different DNA constructs.** 20 µl of culture supernatant were directly analyzed by SDS-PAGE and Coomassie blue staining. M, molecular weight markers; from top to bottom: 180, 130, 100, 70(*), 55, 40, 35, 25, 15, 10kDa.

Expression constructs obtained by R/L cloning provided detectable levels of GH45 and to a lesser extent of GH5, but no protein band was visible at the expected migration position of GH11 (Figure 1, pBGP1). By contrast, expression constructs obtained by Gateway cloning failed to provide any detectable protein on gel with a migration pattern of non transformed cells (Figure 1, pBGP1-DEST; the ≈100kDa band in the three GH45 lanes is a yeast endogenous protein). Since the only difference between pBGP1-DEST and pBGP1 is the presence of in-frame Gateway recombination sites attb1 and attb2 in pBGP1-DEST, we assume that 5’ (attb1) and/or 3’ (attb2) Gateway recombination sites interfere with the translational or post-translational processing of these proteins, which is not in agreement with the data reported in [2]. Nevertheless, the lack of detectable recombinant protein essentially disqualified pBGP1-DEST for our specific use. In conclusion, we decided to use R/L cloning but not Gateway cloning in subsequent experiments despite the obvious advantage of the latter in terms of speed and convenience.

**References.**

[1] Esposito D, Gillette WK, Miller DA, Taylor TE, Frank PH, Hu R, Bekisz J, Hernandez J, Cregg JM, Zoon KC, Hartley JL: [**Gateway cloning is compatible with protein secretion from Pichia pastoris.**](http://www.ncbi.nlm.nih.gov/pubmed/15766886) *Protein Expr Purif* 2005, **40:**424-8.

[2] Sasagawa T, Matsui M, Kobayashi Y, Otagiri M, Moriya S, Sakamoto Y, Ito Y, Lee CC, Kitamoto K, Arioka M: **High-throughput recombinant gene expression systems in Pichia pastoris using newly developed plasmid vectors.** *Plasmid* 2011, **65:**65–69.

[3] Lee CC, Williams TG, Wong DW, Robertson GH: **An episomal expression vector for screening mutant gene libraries in Pichia pastoris.** *Plasmid* 2005, **54:**80–85.

[4] Couturier M, Feliu J, Haon M, Navarro D, Lesage-Meessen L, Coutinho PM, Berrin JG: **A thermostable GH45 endoglucanase from yeast: impact of its atypical multimodularity on activity.** *Microb Cell Fact* 2011, **10:**103.

[5] Couturier M, Haon M, Coutinho PM, Henrissat B, Lesage-Meessen L, Berrin JG. **Podospora anserina hemicellulases potentiate the Trichoderma reesei secretome for saccharification of lignocellulosic biomass**. *Appl Environ Microbiol* 2011, **77:**237-46.
