# Supplementary material for: Recombinant protein production facility for fungal biomass-degrading enzymes using the yeast Pichia pastoris
Source: Front Microbiol. 2015 Sep 23;6:1002. doi: 10.3389/fmicb.2015.01002 (PMC4585289; doi:10.3389/fmicb.2015.01002)
Supplement: Supplementary file 2 [file Data_Sheet_2.DOCX]

**Additional file 2: PCR products generate non expressing *P. pastoris* clones**

After the coding sequence of interest has been inserted in a *P. pastoris* expression vector, the resulting circular construct must be linearized to increase its ability to integrate the genome by homologous recombination if an integrative vector is used. This is generally achieved by restriction digestion. However, the procedure uses a significant amount of time and DNA (up to 10 µg per transformation according to Invitrogen protocol (pgapz_man.pdf)). These two issues have been collectively addressed by using PCR to both linearize and amplify expression constructs. In addition, the use of PCR allows combining a single coding sequence with different upstream and downstream sequences as described below. Two kinds of PCR products have been used in published studies (Figure 1A). The first one is a single fragment obtained by overlapping PCR using three (5’ (promoter), internal (coding sequence) and 3’ (selection marker)) megaprimers [1]. More recently, the procedure was even further simplified: the second PCR (extension of overlapping megaprimers) was omitted and *P. pastoris* cells were transformed with the three megaprimers [2]. We tested both options with GH5 coding sequence.

**Methods.**

Taq (screening purpose) and Elongase (cloning purpose) DNA polymerases (Invitrogen) were used in PCR experiments. PCR products were purified using NucleoSpin^®^ gel and PCR clean-up kit (Macherey-Nagel).

Linear integrative expression constructs were generated by the megaprimer technique. Three overlapping megaprimers corresponding to the promoter region (5’ megaprimer using primers AGATCTTTTTTGTAGAAATGTCTTGG and CTCGAGAAAAGAGAGGCTGAAGCTGAATTC), the coding sequence (using primers CTCGAGAAAAGAGAGGCTGAAGCTGAATTCctcccccaagcacaaggtgga

and CTCTTCTGAGATGAGTTTTTGTTCTAGcgccgggagagcattgatagc)

and the selection marker (3’ megaprimer using primers TCTAGAACAAAAACTCATCTCAGAAGAG and TCTCCAGCTTGCAAATTAAAGCC) were separately generated (PCR1, Figure 1A). To avoid false positive results due to possible inefficient DpnI treatment post-PCR, GH5 coding sequence (1020 bp) was not PCR amplified using GH5-pGAPZαA construct as template but a non expressing Gateway construct (an entry clone resulting from a BP reaction). For the same reason, 5’ (promoter, 700 bp) and 3’ (Zeocin resistance gene, 1600 bp) megaprimers were separately PCR amplified using an empty pGAPZαA vector as template. PCR conditions were [94°C for 5’] x 1, [94°C for 30”, 55°C for 30”, 68°C for 1’ per 1000 bases] x 25, [68°C for 10’] x 1.

The full length linear expression construct was then reconstituted by overlapping PCR (PCR2, Figure 1A). After DpnI treatment to remove the template and purification, 1 µl of each megaprimer was used with the outer primers in a 25 cycles PCR experiment.

PCR screening of *P. pastoris* colonies for the presence of integrated PCR products.

Individual colonies were picked-up from Zeocin agar paltes using tooth-picks, resuspended in 20µl of 20 mM NaOH, and then heated for 10’ at 98°C. One microliter of this mixture was used as template in PCR experiments.

**Figure 1. Linear expression constructs made using PCR**. A, General strategy. B, Migration pattern of the three megaprimers. M: 3, 2, 1.5, 1^(+)^, .75, .5, .25 kb. Lane 1, promoter; lane 2, GH5 coding sequence; lane 3, Zeocin resistance gene. C, full length construct reconstitution by overlapping PCR. M, same as B. Lane 1, positive control using GH5-pGAPZαA as PCR template. Lane 2, reconstituted full length contig. D, PCR screening of two independent yeast clones (clones 1 (lanes 1-3) and 2 (lanes 4-6)) for the presence of each megaprimer in the yeast genome. The primer pairs used amplified the promoter (lanes 1 and 4), or GH5 coding sequence (lanes 2 and 5) or the Zeocin resistance gene (lanes 3 and 6). M, same as B. E, the same two clones as in D were PCR screened using the 5’ primer (denoted by * in A) of the promoter sequence and the 3’ primer (denoted by ** in A) of GH5 coding sequence (1720 bp). M, same as B.

**Results and discussion.**

PCR products were generated as described in Methods. Three overlapping megaprimers respectively corresponding to the promoter region (5’ megaprimer), the coding sequence (internal megaprimer) and the selection marker (3’ megaprimer) were separately generated (Figure 1A (PCR1) and B), and the full length contig was reconstituted by overlapping PCR of the three megaprimers (Figure 2A (PCR2) and C). Equal molar amounts of the full length contig or of a mix of the three megaprimers were used to electroporate X33 cells. Recombinant clones were selected on Zeocin agar plates, and the presence of the construct in the yeast genome was checked by PCR for two of them that had been transformed with the three megaprimers mix (Figure 1D). The 5’ (promoter) and internal (GH5) fragments were definitely amplified, but the 3’ fragment (Zeocin resistance gene) was not. However, since the clones had been selected on Zeocin agar plates, this negative result was likely to be a false negative. Detecting individual DNA fragments does not guaranty that they are correctly ordered to generate the expected contig. To check this point, another PCR screen was performed using primers respectively located by * and ** in Figure 1A. No attempt was made to amplify the full length construct since the 3’ primer of the Zeocin resistance gene was seemingly unable to hybridyze with its complementary sequence once integrated in the yeast genome (Figure 1D, lanes 3 and 6). A fragment of expected size (1720 bp) corresponding to the correct contig made of the 5’ (promoter) followed by the internal (GH5) fragments was detected in the genome of clones 1 and 2 (Figure 1E), indicating that transforming yeast cells with overlapping DNA fragments resulted in the integration of the reconstituted contig in the genome. The screening was not performed on yeast clones that had been transformed with the full length fragment (PCR2 product in Figure 1A) because this was redundant with the Zeocin selection.

Unfortunately, none of the ten tested clones expressed GH5 (Figure 2, PCR 3 megaprimers), whereas GH5 was expressed by three independent clones that had been transformed by a GH5-pGAPZαA construct obtained by R/L and linearized by restriction (Figure 2, pGAPZαA).

**Figure 2. Expression results of PCR products.** 10 µl of culture supernatant of cells that had been transformed with the three megaprimers or with pGAPZaA bearing GH5 coding sequence were directly analyzed by SDS-PAGE. M, molecular weight markers. From top to bottom: 180, 130, 100, 70, 55(*), 40, 35, 25, 15, 10kDa.

This suggests that despite correct integration in the yeast genome (Figure 1E), overlapping PCR is not the most reliable DNA transfer technique, at least in our hands for GH5 and for our goal of setting-up a recombinant protein production facility at lab scale.

In conclusion, constructs used subsequently in this study were amplified in *E. coli* and linearized by restriction.

**References.**

[1] Liu Z, Pscheidt B, Avi M, Gaisberger R, Hartner FS, Schuster C, Skranc W, Gruber K, Glieder A: **Laboratory evolved biocatalysts for stereoselective syntheses of substituted benzaldehyde cyanohydrins.** *ChemBioChem* 2008, **9:**58–61.

[2] Yu XW, Wang R, Zhang M, Xu Y, Xiao R: **Enhanced thermostability of a Rhizopus chinensis lipase by in vivo recombination in Pichia pastoris.** *Microb Cell Fact* 2012, **11:**1022.
