# Supplementary material for: Recombinant protein production facility for fungal biomass-degrading enzymes using the yeast Pichia pastoris
Source: Front Microbiol. 2015 Sep 23;6:1002. doi: 10.3389/fmicb.2015.01002 (PMC4585289; doi:10.3389/fmicb.2015.01002)
Supplement: Supplementary file 3 [file Data_Sheet_3.DOCX]

**Additional file 3: fractional factorial approach of GH11 expression**

**Method.**

When different values of different parameters are combined in a factorial approach, the full factorial is made of all the combinations of all the values of all the parameters. Since the same number of values and parameters were used for pGAPZαA and pPICZαA (see tables below) the full factorial amounted to 3 x 3 x 4 = 36 combinations in both cases. For reducing this number down to a manageable number of experiments while keeping the approach meaningful, we used online SAmBA software (<http://www.igs.cnrs-mrs.fr/samba/>) to generate a fractional factorial approach using 12 out of 36 combinations of the full factorial.

1. pGAPZαA

| Parameter | Temperature | pH | Carbon source |
| --- | --- | --- | --- |
| Value | 27°C | 9 | glucose |
|  | 18°C | 6 | glycerol |
|  | 10°C | 3 | saccharose |
|  |  |  | sorbitol |

The fractional factorial was designed in such a way that it fits with 24-wells deepwell plates format:

DW 27°C:

| Protein 1 | Glucose / pH3 | Glycerol / pH9 | Saccharose / pH3 | Sorbitol / pH6 |
| --- | --- | --- | --- | --- |
| Protein 2 | Glucose / pH3 | Glycerol / pH9 | Saccharose / pH3 | Sorbitol / pH6 |
| Protein 3 | Glucose / pH3 | Glycerol / pH9 | Saccharose / pH3 | Sorbitol / pH6 |
| Protein 4 | Glucose / pH3 | Glycerol / pH9 | Saccharose / pH3 | Sorbitol / pH6 |

DW 18°C:

| Protein 1 | Glucose / pH9 | Sorbitol / pH9 | Saccharose / pH6 | Glycerol / pH3 |
| --- | --- | --- | --- | --- |
| Protein 2 | Glucose / pH9 | Sorbitol / pH9 | Saccharose / pH6 | Glycerol / pH3 |
| Protein 3 | Glucose / pH9 | Sorbitol / pH9 | Saccharose / pH6 | Glycerol / pH3 |
| Protein 4 | Glucose / pH9 | Sorbitol / pH9 | Saccharose / pH6 | Glycerol / pH3 |

DW 10°C:

| Protein 1 | Glycerol / pH6 | Glucose / pH6 | Saccharose / pH9 | Sorbitol / pH3 |
| --- | --- | --- | --- | --- |
| Protein 2 | Glycerol / pH6 | Glucose / pH6 | Saccharose / pH9 | Sorbitol / pH3 |
| Protein 3 | Glycerol / pH6 | Glucose / pH6 | Saccharose / pH9 | Sorbitol / pH3 |
| Protein 4 | Glycerol / pH6 | Glucose / pH6 | Saccharose / pH9 | Sorbitol / pH3 |
|  |  |  |  |  |

1. pPICZαA

| Parameter | Temperature | pH | Methanol (%) |
| --- | --- | --- | --- |
| Value | 27°C | 9 | 10 |
|  | 18°C | 6 | 3 |
|  | 10°C | 3 | 1 |
|  |  |  | 0.1 |

The fractional factorial was designed in such a way that it fits with 24-wells deepwell plates format:

DW 27°C:

| Protein 1 | M3 / pH9 | M0.1 / pH9 | M1 / pH6 | M10 / pH3 |
| --- | --- | --- | --- | --- |
| Protein 2 | M3 / pH9 | M0.1 / pH9 | M1 / pH6 | M10 / pH3 |
| Protein 3 | M3 / pH9 | M0.1 / pH9 | M1 / pH6 | M10 / pH3 |
| Protein 4 | M3 / pH9 | M0.1 / pH9 | M1 / pH6 | M10 / pH3 |

DW 18°C:

| Protein 1 | M10 / pH6 | M3 / pH6 | M0.1 / pH3 | M1 / pH9 |
| --- | --- | --- | --- | --- |
| Protein 2 | M10 / pH6 | M3 / pH6 | M0.1 / pH3 | M1 / pH9 |
| Protein 3 | M10 / pH6 | M3 / pH6 | M0.1 / pH3 | M1 / pH9 |
| Protein 4 | M10 / pH6 | M3 / pH6 | M0.1 / pH3 | M1 / pH9 |

DW 10°C:

| Protein 1 | M0.1 / pH6 | M3 / pH3 | M1 / pH3 | M10 / pH9 |
| --- | --- | --- | --- | --- |
| Protein 2 | M0.1 / pH6 | M3 / pH3 | M1 / pH3 | M10 / pH9 |
| Protein 3 | M0.1 / pH6 | M3 / pH3 | M1 / pH3 | M10 / pH9 |
| Protein 4 | M0.1 / pH6 | M3 / pH3 | M1 / pH3 | M10 / pH9 |

Note. “M” stands for methanol concentration. For example, “M3” means 3% methanol.

**Detailed analysis of the results reported in Figure 6.**

The three variables tested with pGAPZαA were the temperature (27°C, 18°C, 10°C), the pH (3, 6, 9) and the carbon source (glucose, glycerol, saccharose, sorbitol). Reference medium YPD was included as control at all three temperatures. Results (Figure 6A) indicated that expression was proportional to the temperature, and that an alkaline pH was more favourable than an acidic one. However, the deleterious effect of the most acidic condition (pH3) could not be compensated by increasing the temperature. The best expression medium was the reference YPD the pH of which was about 8 at the end of the expression period. This positive effect of alkaline pH was somewhat surprising considering the theoretical alkaline pI of GH11 (8.84). It could be related either to the inactivation of acidic proteases [Sinha *et al*., 2005] or to the fact that YPD is slightly acidic at the beginning of culture / expression period and becomes slightly alkaline only as a result of biomass growth. For an unknown reason, YPD lost its leadership when expression temperature decreased. As to the third variable, glucose and glycerol proved better carbon sources than saccharose or sorbitol.

Two of the three variables tested with pPICZαA were the same as those used with pGAPZαA (pH and temperature), but the carbon source was replaced with the percentage of methanol used for inducing expression (0.1, 1, 3, 10%). Results are reported in Figure 6B. Protein band intensities and enzymatic activities were roughly in the same range as those obtained with pGAPZαA. Expression from pPICZαA was also proportional to the temperature, and was even lower at 10°C than that obtained with pGAPZαA. This experiment also confirmed that almost no expression (protein band in gel or enzymatic activity) was detected at pH3. Best methanol concentration was 1 to 3%, with no possibility to distinguish between these two values since identical results were obtained with 1 and 3% methanol at the same pH (6) and temperature (27°C) (compare lanes 3 and 5 of Figure 6B). By contrast, extreme values (0.1% (compare lanes 1 and 2) and 10% (compare lanes 6 and 7)) were poorer inducers. On this basis, interesting information on the role of expression pH came from comparison of the first three lanes of Figure 6B. *i*) Whereas the enzymatic activity was roughly the same in lanes 2 and 3, protein band intensity in lane 2 (M0.1 pH9) accounted for definitely less protein than in lane 3 (M1 pH6), presumably because not enough inducer was used in the former (M0.1). In other words, GH11 specific activity was higher at pH9 (lane 2) than at pH6 (lane 3). *ii*) The same conclusion could be drawn from comparison of lanes 1 and 3: for roughly the same amount of protein, conditions of lane 1 (M3 pH9) provided more activity than those of lane 3 (M1 pH6) for methanol concentrations considered equally potent (M3 and M1, respectively). The same reasoning could be applied to the influence of expression temperatures: reducing temperature from 27°C (lane 5) to 18°C (lane 7) led the same combination of pH (6) and methanol concentration (3%) to provide slightly less protein with more enzymatic activity. Comparison of lanes 5 and 7 therefore suggested that reducing expression temperature from 27°C to 18°C increased GH11 specific activity as did raising pH from 6 to 9, presumably by improving GH11 folding or even the whole post-translational process in both cases. Improvement of post-translational processing by dropping expression temperature from 30°C to 20°C was also reported for the human interleukin 10 expressed in *P. pastoris* [**Zhong *et al*., 2014**].

Testing different expression conditions was easy: although experiments reported in Figure 6 required performing 87 independent cultures, use of 24-wells deepwell plates only amounted to a single plate per expression temperature. In addition, the good reproducibility among culture triplicates (band intensities and enzymatic activity standard deviations) created a reliable basis for comparing the effects of varying several parameters at a time. Finally, even if they did not allow increasing GH11 expression over that provided by the basic conditions, the two fractional factorial approaches allowed pointing out in a single experiment the requests of GH11 specific activity in terms of pH and temperature.

**References.**

Sinha, J., Plantz, B. A., Inan, M., and Meagher, M. M. (2005) Causes of proteolytic degradation of secreted recombinant proteins produced in methylotrophic yeast Pichia pastoris: case study with recombinant ovine interferon-T. *Biotechnol Bioeng*. 89, 102-112.

# **Zhong,** **Y.**, **Yang ,** **L.**, **Guo,** **Y.**, **Fang,** **F.**, **Wang,** **D.**, **Li,** **R.**, *et al*. (2014) High-temperature cultivation of recombinant Pichia pastoris increases endoplasmic reticulum stress and decreases production of human interleukin-10. Microb Cell Fact. **13**, 163.
